# Long non-coding RNA EGOT is associated with ^131^iodine sensitivity and contributes to thyroid cancer progression by targeting miR-641/PTEN axis

**DOI:** 10.18632/aging.205284

**Published:** 2023-11-21

**Authors:** Ming Wang, Zhengchao Wei, Shuang Wang, Wenjuan Feng, Lihua Shang, Xiaosong Sun

**Affiliations:** 1 Department of Thyroid-Head and Neck Oncosurgery-1, Jilin Cancer Hospital, Changchun, Jilin Province, China; 2 Department of Thoracic Oncosurgery-1, Jilin Cancer Hospital, Changchun, Jilin Province, China; 3 Department of Gynecologic Oncosurgery-2, Jilin Cancer Hospital, Changchun, Jilin Province, China; 4 Department of Breast Oncosurgery-2, Jilin Cancer Hospital, Changchun, Jilin Province, China

**Keywords:** thyroid cancer, 131I resistance, lncRNA EGOT, miR-641, PTEN, clinical functions

## Abstract

Thyroid cancer is a prevalent endocrine malignancy around the world. Radioactive ^131^iodine (^131^I) therapy is widely applied in TC patients, but the resistance affects its effectiveness in the clinics. Long non-coding RNA (lncRNA) EGOT has been reported to induce an inhibitory effect on cancer progression, but the specific function of EGOT in ^131^I resistance of TC cells remains unclear. Here, we successfully established ^131^I-resistant TC cells and evaluated the impact of EGOT on ^131^I resistance in the cells. Our data showed that EGOT and PTEN expression was reduced but the miR-641 expression was enhanced in ^131^I-resistant TC cells. EGOT inhibited viability, induced apoptosis and enhanced DNA damage in ^131^I-resistant TC cells. Mechanically, we identified that EGOT induced PTEN expression by targeting miR-641 in 131I-resistant TC cells. Moreover, the depletion of PTEN and miR-641 mimic reversed EGOT-relieved ^131^I resistance of TC cells *in vitro*. Thus, we conclude that lncRNA EGOT attenuated ^131^I resistance of TC cells by targeting miR-641/PTEN axis. The clinical functions of EGOT in TC therapy deserve to be validated in future exploration.

## INTRODUCTION

Thyroid cancer (TC) is the most prevalent endocrine malignancy around the world, especially in women, and shows an increasing trend annually [1, 2]. At present, the operative treatment is the standard therapy for TC patients [3]. In addition to surgical method, radioactive iodine (^131^I) therapy is also widely applied in those patients with advanced follicular cell-derived TC, to potentially eradicate the residual lesions after surgery [4]. However, some patients would show ^131^I resistance due to local recurrence and distance metastasis or other reasons [3]. Hence, it is urgent to develop effective adjuvant therapeutic strategies for patients with ^131^I resistance.

Over the past decades, the discovery of noncoding RNAs has greatly improved the development of cancer research. Among which, long noncoding RNAs (lncRNAs) are defined as a class of RNAs with a sequence longer than 200 nucleotides, and are involved in almost every step of carcinogenesis through functioning as miRNA sponges and participates in the ceRNA network [5]. The participation of lncRNAs in therapeutic resistance is also widely studied [6, 7]. Eosinophil granule ontogeny transcript (EGOT) is an antisense intronic long noncoding RNA expressed by ITPR1, whose function remains unclarified [8]. A recent study suggested that EGOT could stimulate the sensitivity of breast cancer cells to paclitaxel through enhancing autophagosome accumulation [9]. However, the connection between EGOT with thyroid cancer, especially the ^131^I resistance, has not been determined yet.

MiRNA is a highly conserved type of endogenous noncoding RNA, closely related to cancer progression via targeting numerous mRNAs and interfering gene expression [10]. MiR-641 is a miRNA that functions differently upon varied cancer context. It is reported to act as an activator of ERK signaling through directly targeting NF1, and facilitates the erlotinib resistance of NSCLS cells [11]. A recent study indicated that miR-641 is involved in PTEN-related migration and invasion of cervical cancer cells [12]. However, the function of miR-641 in TC is still elusive. PTEN is a representative tumor suppressor, and was found to be low expressed in various cancers including thyroid cancer [13]. The loss of PTEN is associated with therapeutic resistance of several cancers [14, 15]. However, the direct connection between miR-641 and PTEN in thyroid cancer is still not clear.

In this work, we successfully established ^131^I resistant thyroid cancer cell lines, and observed a notably declined level of lncRNA EGOT in ^131^I resistant cells. Mechanically, we proposed that EGOT functioned as a sponge of miR-641, suppressed cell viability, enhanced apoptosis and DNA damage. Further study identified tumor suppressor PTEN as a target of miR-641, which established an EGOT/miR-641/PTEN regulatory axis in ^131^I resistant thyroid cancer. Our work provided a new perspective and potential target for the therapy of ^131^I resistant thyroid cancer.

## RESULTS

### The establishment of ^131^I-resistant TC cells

**Figure 1:**
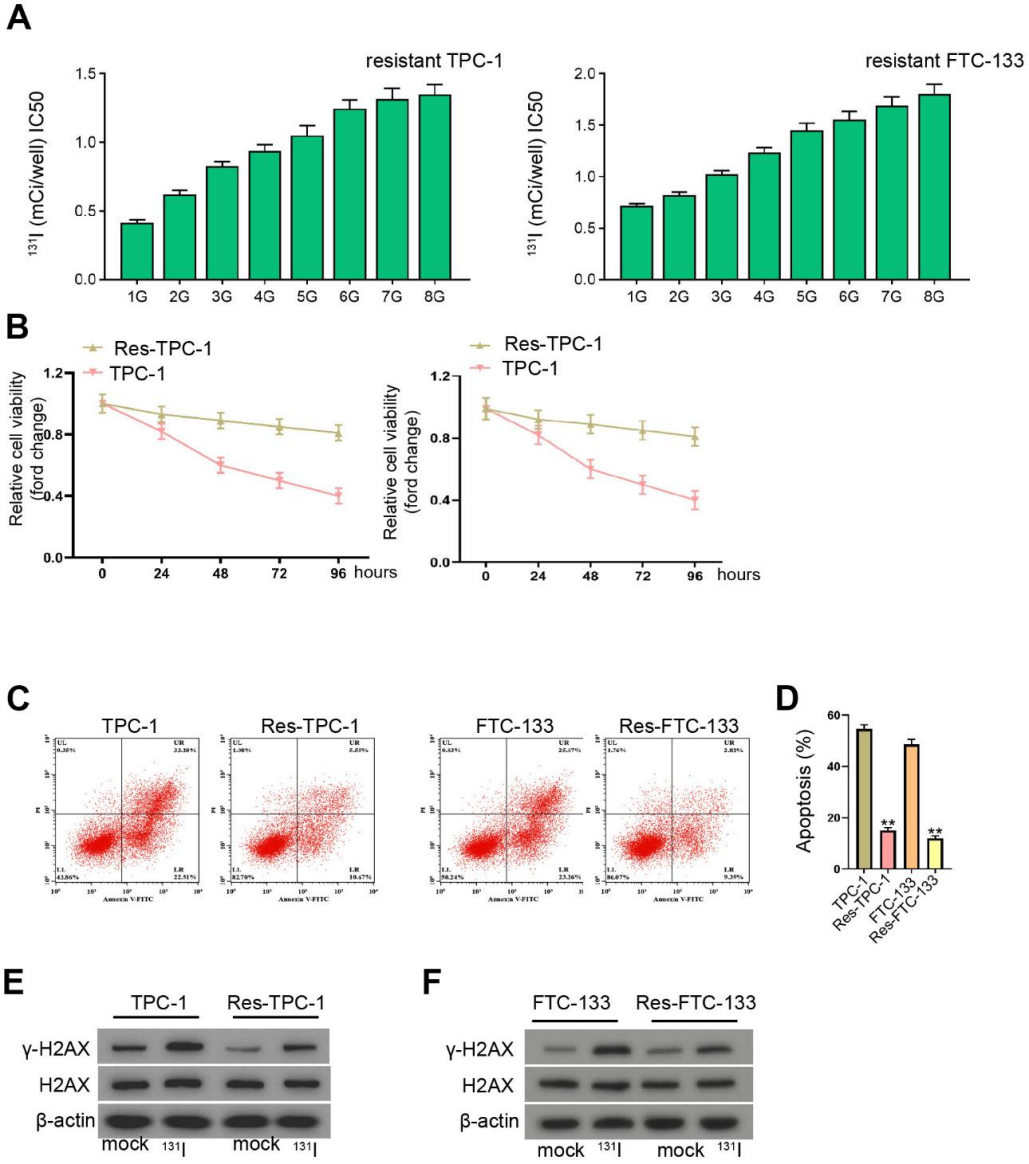
**The establishment of 131I-resistant TC cells.** (**A**–**F**) TPC-1 and FTC-133 cells were treated with sub-lethal ^131^I. (**A**) The ^131^I -resistant TPC-1 and FTC-133 cells were established after 8-continuous passages. (**B**) The cell viability was measured by CCK-8 assays in TPC-1, FTC-133 cells, and ^131^I -resistant TPC-1 and FTC-133 cells. (**C**, **D**) Flow cytometry analysis of cell apoptosis in the cells. (**E**, **F**) Western blot analysis of γ-H2AX expression in the cells. mean ± SD, ** *P* < 0.01. Experiments were repeated at least biological triplicates.

To evaluate the effect of lncRNA EGOT on ^131^I resistance of TC cells, we constructed the ^131^I-resistant TC cells by continuously exposing FTC-133, TPC-1, and BCPAP cells with ^131^I. After continuous passage for 8 generations, ^131^I-resistant TC cells were successfully constructed, with increase of 131I median lethal intensity compared with ^131^I-sensitive FTC-133 and TPC-133 cells, as demonstrated the IC50 of ^131^I using CCK-8 assay (Figure 1A and Supplementary Figure 1A). The cell viability of ^131^I-resistant TPC-1 and FTC-133 cells was significantly enhanced compared with TPC-1 and FTC-133 cells (Figure 1B). As expected, ^131^I-resistant FTC-133, TPC-1, and BCPAP cell apoptosis were significantly repressed compared with FTC-133, TPC-1, and BCPAP cells, in which ^131^I treatment inhibited the cell apoptosis (Figure 1C, 1D, and Supplementary Figure 1B). In addition, the expression DNA damage marker γ-H2AX was inhibited in ^131^I-resistant FTC-133, TPC-1, and BCPAP cells relative to FTC-133, TPC-1, and BCPAP cells, while the treatment of 131I (0.5 mCi) enhanced γ-H2AX expression (Figure 1E, 1F, and Supplementary Figure 1C).

### EGOT and PTEN expression is decreased and miR-641 expression is increased in ^131^I-resistant TC cells

**Figure 2:**
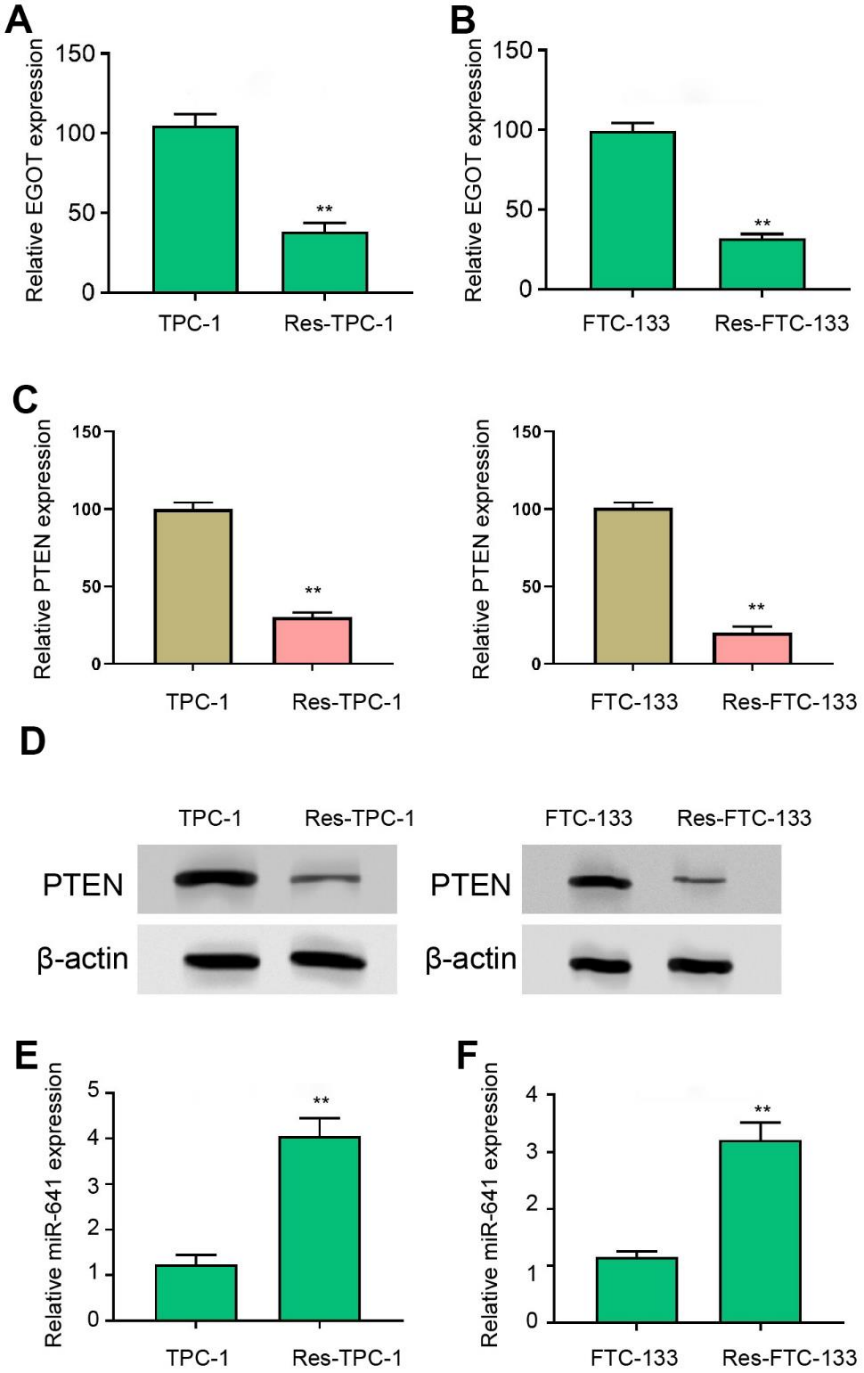
**EGOT and PTEN expression is decreased and miR-641 expression is increased in 131I-resistant TC cells.** (**A**, **B**) The qPCR analysis of EGOT in ^131^I -resistant TPC-1 and FTC-133 cells. (**C**) The qPCR analysis of PTEN in ^131^I -resistant TPC-1 and FTC-133 cells. (**D**) The Western blot analysis of PTEN in ^131^I -resistant TPC-1 and FTC-133 cells. (**E**, **F**) The qPCR analysis of miR-641 in ^131^I -resistant TPC-1 and FTC-133 cells. mean ± SD, ** *P* < 0.01. Experiments were repeated at least biological triplicates.

Then, we detected the expression of EGOT, miR-641, and PTEN in ^131^I-resistant TPC-1 and FTC-133 cells. We found that the EGOT and PTEN expression was reduced but miR-641 expression was enhanced in ^131^I-resistant FTC-133, TPC-1, and BCPAP cells compared with TPC-1 and FTC-133 cells (Figure 2 and Supplementary Figure 2A–2C). Meanwhile, the expression of EGOT and PTEN was reduced but miR-641 expression was enhanced in the clinical TC tissues (Supplementary Figure 2D).

### EGOT represses viability, enhances apoptosis and induces DNA damage in ^131^I-resistant TC cells

**Figure 3:**
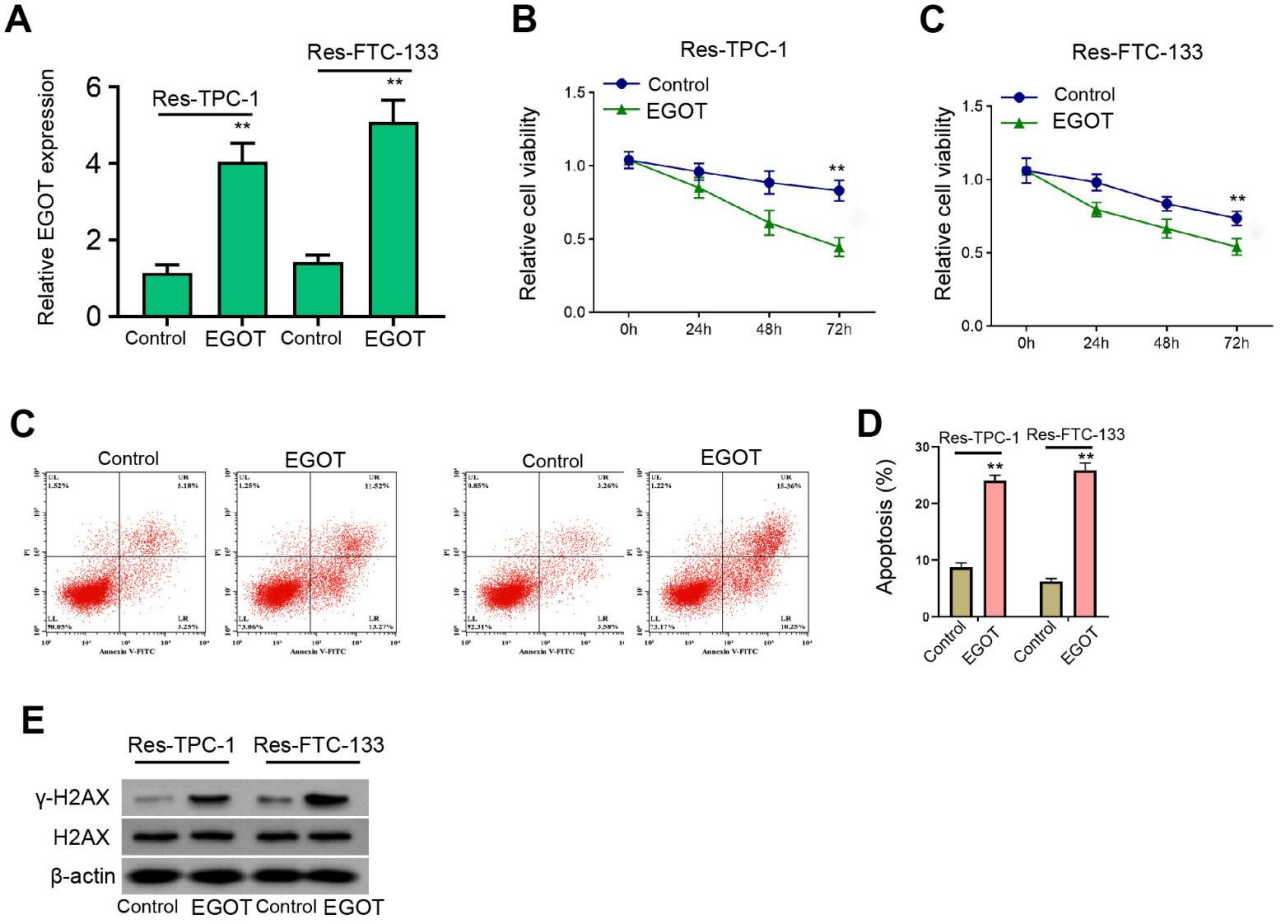
**EGOT represses viability, enhances apoptosis and induces DNA damage in 131I-resistant TC cells.** (**A**–**E**) The ^131^I -resistant TPC-1 and FTC-133 cells were treated with EGOT overexpression vectors. (**A**) The qPCR analysis of EGOT in the cells. (**B**, **C**) CCK-8 analysis of cell viabilities. (**D**) Flow cytometry analysis of cell apoptosis in the cells. (**E**) Western blot analysis of γ-H2AX expression in the cells. mean ± SD, ** *P* < 0.01. Experiments were repeated at least biological triplicates.

Next, we analyzed the effect of EGOT on malignant phenotypes of ^131^I-resistant TPC-1 and FTC-133 cells. The ^131^I-resistant FTC-133, TPC-1, and BCPAP cells were transfected with EGOT overexpression vectors and the efficiency was confirmed in the cells (Figure 3A and Supplementary Figure 3A). Meanwhile, the overexpression of EGOT reduced ^131^I-resistant FTC-133, TPC-1, and BCPAP cell viabilities compared with the control plasmid (Figure 3B, 3C, and Supplementary Figure 3B). The ^131^I-resistant FTC-133, TPC-1, and BCPAP cell apoptosis were stimulated by the overexpression of EGOT compared with the control plasmid (Figure 3D and Supplementary Figure 3C). The γ-H2AX levels were induced by EGOT in the ^131^I-resistant FTC-133, TPC-1, and BCPAP cells compared with the control plasmid (Figure 3E and Supplementary Figure 3D). Meanwhile, we validated that the overexpression of EGOT repressed the migration and invasion of ^131^I-resistant FTC-133, TPC-1, and BCPAP cells compared with the control plasmid (Supplementary Figure 4A, 4B).

### LncRNA EGOT serves as a sponge of miR-641 to enhance PTEN expression in ^131^I-resistant TC cells

**Figure 4:**
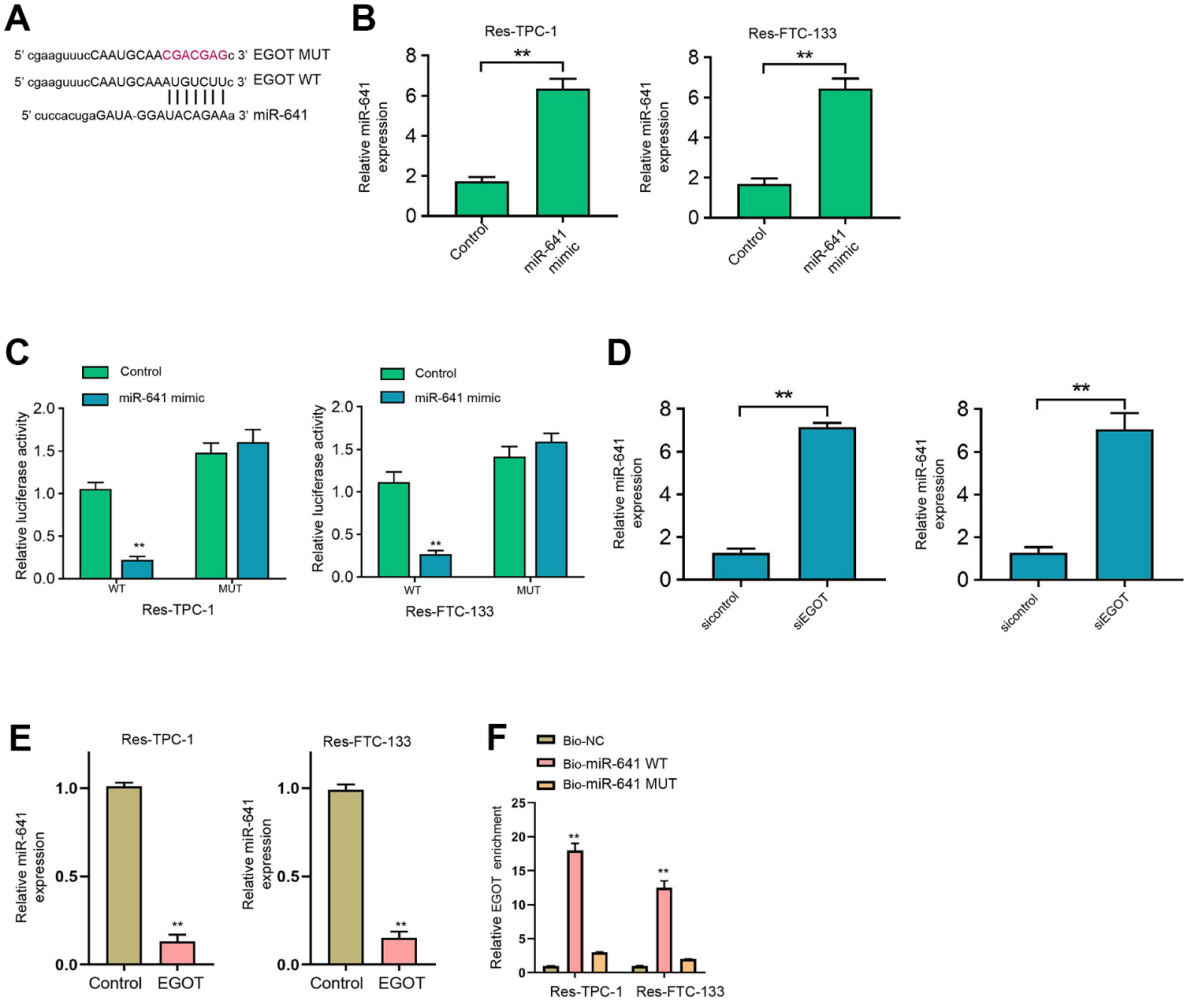
**EGOT is able to sponge miR-641 in 131I-resistant TC cells.** (**A**) The binding side prediction of EGOT and miR-641 in the ENCORI database. (**B**, **C**) The ^131^I -resistant TPC-1 and FTC-133 cells were treated with miR-641 mimic. (**B**) The qPCR analysis of miR-641 in the cells. (**C**) Luciferase reporter gene assays of EGOT luciferase activities. (**D**) The qPCR analysis of miR-641 in ^131^I -resistant TPC-1 and FTC-133 cells treated with EGOT siRNA. (**E**) The qPCR analysis of miR-641 in ^131^I -resistant TPC-1 and FTC-133 cells treated with EGOT overexpressing plasmid. (**F**) The direct interaction of EGOT and miR-641 was analyzed by RNA pull down. mean ± SD, ** *P* < 0.01. Experiments were repeated at least biological triplicates.

We then found the binding side within EGOT and miR-641 in the ENCORI database (Figure 4A). We treated ^131^I-resistant FTC-133, TPC-1, and BCPAP cells with miR-641 mimic and verified the enhanced miR-641 expression in the cells (Figure 4B and Supplementary Figure 5A). The treatment of miR-641 mimic repressed luciferase activity of EGOT, but not EGOT mutant, in ^131^I-resistant FTC-133, TPC-1, and BCPAP cells compared with the control mimic (Figure 4C and Supplementary Figure 5B). The depletion of EGOT induced miR-641 expression in the cells compared with the control shRNA (Figure 4D and Supplementary Figure 5C). Meanwhile, the overexpression of EGOT repressed miR-641 expression in the cells compared with the control plasmid (Figure 4E). RNA pull-down assays identified the direct interaction of EGOT and miR-641 (Figure 4F).

**Figure 5:**
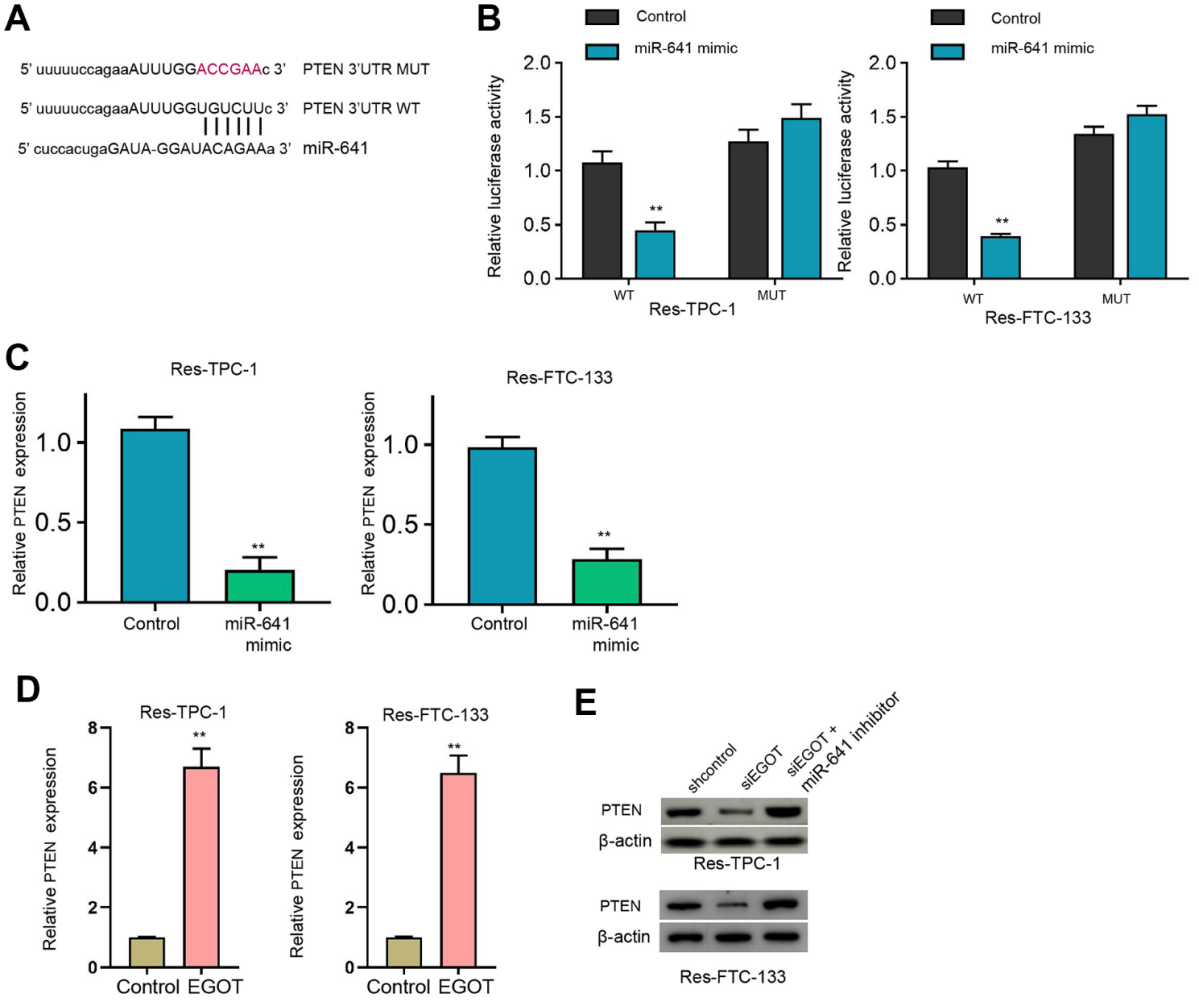
**MiR-641 can target PTEN in ^131^I-resistant TC cells.** (**A**) The binding side prediction of PTEN and miR-641 in the ENCORI database. (**B**, **C**) The ^131^I -resistant TPC-1 and FTC-133 cells were treated with miR-641 mimic. (**B**) Luciferase reporter gene assays of PTEN mRNA 3’UTR luciferase activities. (**C**, **D**) The qPCR analysis of PTEN in the cells. (**E**) The Western blot analysis of miR-641 in ^131^I -resistant TPC-1 and FTC-133 cells treated with EGOT siRNA, or co-treated with EGOT siRNA and miR-641 inhibitor. mean ± SD, ** *P* < 0.01. Experiments were repeated at least biological triplicates.

Moreover, we identified the binding side within PTEN mRNA 3’UTR and miR-641 in the ENCORI database (Figure 5A). The treatment of miR-641 mimic repressed luciferase activity of PTEN mRNA 3’UTR, but not PTEN mRNA 3’UTR mutant, in ^131^I-resistant FTC-133, TPC-1, and BCPAP cells compared with the control mimic (Figure 5B and Supplementary Figure 6A). MiR-641 suppressed PTEN expression in ^131^I-resistant FTC-133, TPC-1, and BCPAP cells compared with the control mimic (Figure 5C and Supplementary Figure 6B). Meanwhile, the overexpression of EGOT enhanced PTEN expression in the cells compared with the control plasmid (Figure 5D). The silencing of EGOT repressed PTEN levels while co-treatment of miR-641 inhibitor reversed this effect in ^131^I-resistant FTC-133, TPC-1, and BCPAP cells (Figure 5E and Supplementary Figure 6C).

**Figure 6:**
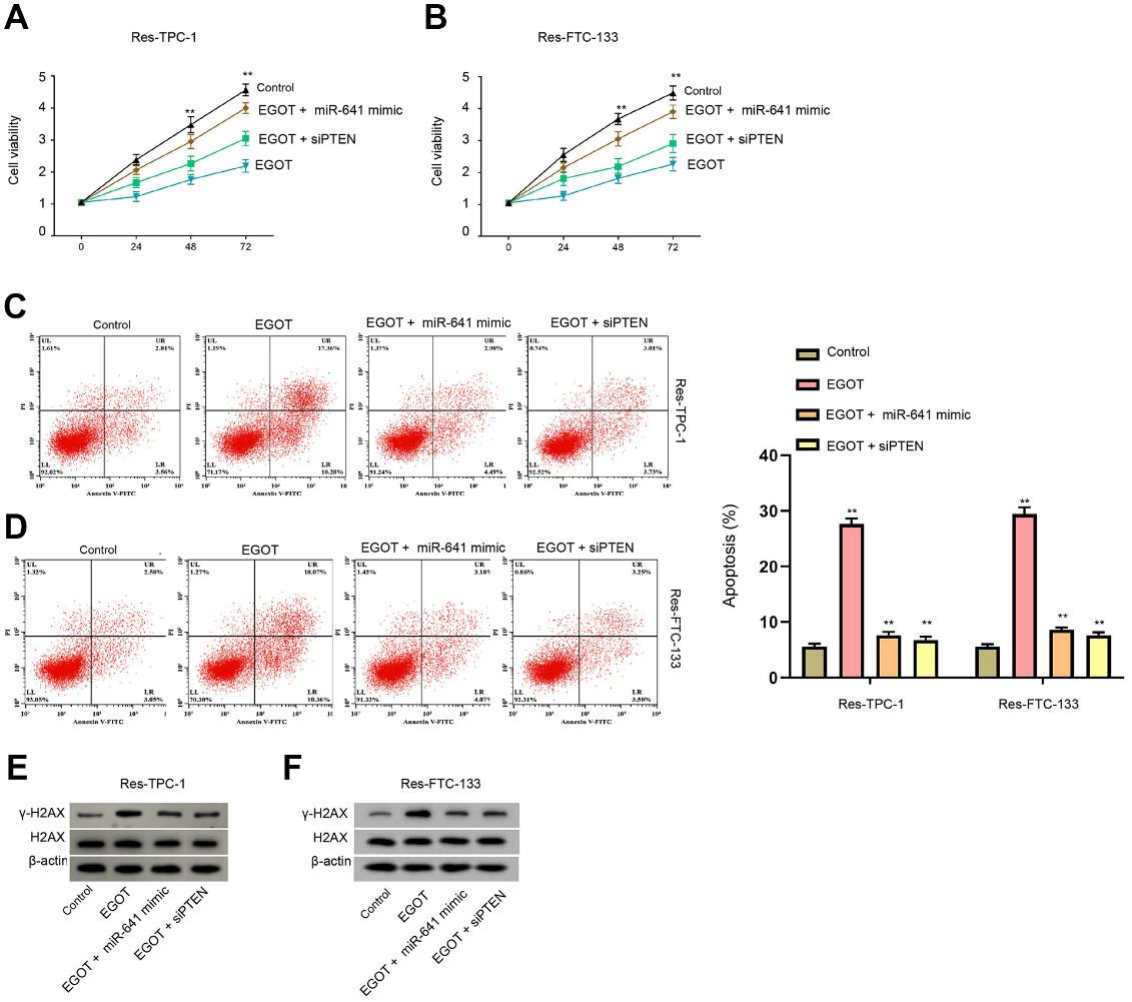
**EGOT regulates viability, apoptosis and DNA damage of 131I-resistant TC cells by targeting miR-641/PTEN axis.** (**A**–**E**) The ^131^I -resistant TPC-1 and FTC-133 cells were treated with EGOT overexpression vectors, or co-treated with EGOT overexpression vectors and PTEN siRNA or miR-641 mimic. (**A**, **B**) CCK-8 analysis of cell viabilities. (**C**, **D**) Flow cytometry analysis of cell apoptosis in the cells. (**E**, **F**) Western blot analysis of γ-H2AX expression in the cells. mean ± SD, ** *P* < 0.01. Experiments were repeated at least biological triplicates.

### EGOT regulates viability, apoptosis and DNA damage of ^131^I-resistant TC cells by targeting miR-641/PTEN axis

We then assess the function of EGOT/miR-641/PTEN axis in the modulation of viability, apoptosis and DNA damage of ^131^I-resistant TC cells. Our data demonstrated that the overexpression of EGOT reduced viabilities and enhanced apoptosis of ^131^I-resistant FTC-133, TPC-1, and BCPAP cells compared with the control plasmid, while miR-641 mimic or PTEN siRNA was able to reverse these phenotypes (Figure 6A–6D and Supplementary Figure 7). Meanwhile, the expression of γ-H2AX was induced by EGOT overexpression in ^131^I-resistant TPC-1 and FTC-133 cells compared with the control plasmid, in which miR-641 mimic or PTEN siRNA could block this impact (Figure 6E, 6F).

**Figure 7:**
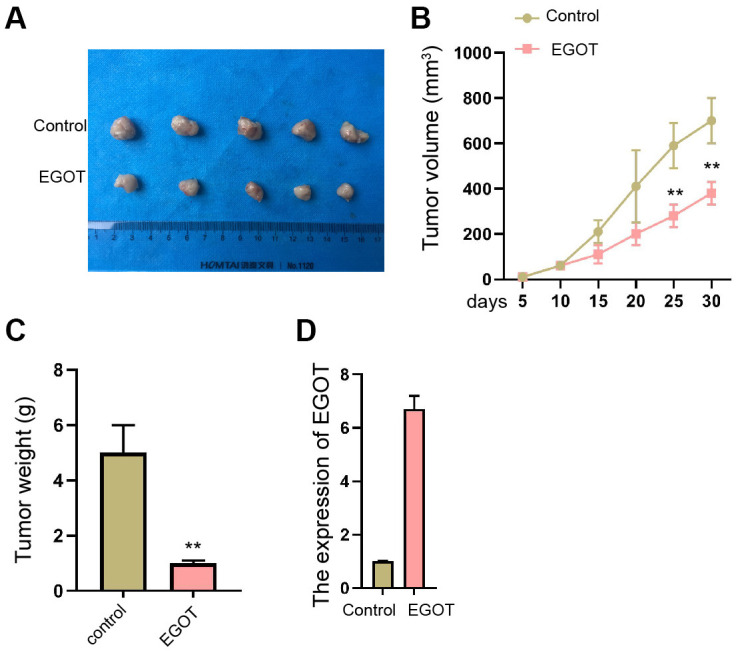
**EGOT suppresses the tumor growth of TC cells *in vivo*.** (**A**–**D**) The effect of EGOT on TC cell tumor growth was analyzed by *In vivo* tumor xenograft in nude mice injected with ^131^I-resistant TPC-1 cells. The tumor images (**A**), tumor volume (**B**), tumor weight (**C**), and EGOT expression (**D**) were shown. mean ± SD, ** *P* < 0.01.

We then validated that the overexpression of EGOT repressed the tumor growth of TC cells in nude mice compared with the control plasmid, as demonstrated by the reduced tumor size, tumor volume, and tumor weight (Figure 7A–7C and Supplementary Figure 7). The overexpression of EGOT was validated in the model (Figure 7D).

## DISCUSSION

TC is endocrine cancer globally with severe morbidity and mobility [3]. Radioactive ^131^I therapy is widely used in TC patients but the ^131^I resistance is a common clinical challenge due to the local recurrence and distance metastasis [3]. Here, we identified the critical function of a lncRNA called EGOT in attenuating ^131^I resistance of TC cells.

Previous studies have reported several lncRNAs in affecting ^131^I resistance of TC cells. NEAT1 inhibition relives ^131^I resistance of TC cells by miR-101-3p/FN1/PI3K/AKT signaling [16]. LncRNA MEG3 contributes to ^31^I I sensitivity by targeting miR-182 in thyroid carcinoma [17]. LncRNA CASC2 represses ^131^I resistance of TC cells via sponging miR-155 [18]. Meanwhile, it has been reported that lncRNA EGOT sensitizes paclitaxel cytotoxicity via enhancing autophagy by upregulating ITPR1 expression in cancer cells [9]. LncRNA EGOT modulates apoptosis and proliferation by the miR-33b-5p/CROT signaling in colorectal cancer [19]. LncRNA EGOT represses migration and viability of breast cancer cells by targeting Hedgehog signaling [20]. LncRNA EGOT serves as the tumor inhibitor of renal cell carcinoma cells [21]. These reports indicate that EGOT may serve as a tumor suppressor in cancer development, but the function of EGOT in TC remains unclear. In this study, we successfully established ^131^I resistant TC cells and identified a decreased expression of EGOT and PTEN and increased miR-641 expression in ^131^I resistant TC cells. Meanwhile, EGOT inhibited viability, induced apoptosis and enhanced DNA damage in ^131^I resistant TC cells. Our data provide an innovative function of lncRNA EGOT in TC and present the effect of EGOT on ^131^I resistant TC cells, demonstrating a new regulatory mechanism of the regulation of ^131^I resistance in TC. Interestingly, the treatment of ^131^I (0.5 mCi) enhanced γ-H2AX expression, but the γ-H2AX was inhibited in ^131^I -resistant TC cell lines, which was consistent with the previous reports [17].

Moreover, it has been reported that the up-regulation of miR-641 induces erlotinib resistance by inhibiting NF1 in non-small-cell lung cancer [11]. LncRNA LINC01963 represses pancreatic carcinoma progression via regulating miR-641/TMEFF2 axis [22]. LncRNA TUSC8 suppresses cervical cancer cell migration/invasion by miR-641/PTEN signaling [12]. These reports indicate that miR-641 is associated with the drug resistance and may serve as a contributor to cancer development. In addition, the correlation of PTEN malignant progression of TC was widely reported. The inhibition of Elk1 represses TC development by modulating PTEN expression [23]. MiR-17-5p contributes to autophagy and proliferation and inhibits apoptosis of TC cells by targeting PTEN [24]. LINC00893 decreases TC progression by regulating AKT pathway through controlling PTEN [25]. In the present study, we identified that EGOT induced PTEN expression by targeting miR-641 in ^131^I resistant TC cells. The depletion of PTEN and miR-641 mimic reversed EGOT-relieved ^131^I resistance of TC cells *in vitro*. These data provide new evidence that miR-641 serves as an important factor in ^131^I resistance of TC cells. The specific function of miR-641 in TC progression is needed to be further investigated in the future. Meanwhile, our data provide new insights into the mechanism involving EGOT, miR-641, and EGOT in TC cells.

In conclusion, we discovered that lncRNA EGOT is associated with ^131^I resistance and contributed to TC progression by targeting miR-641/PTEN axis. The clinical functions of EGOT in TC therapy are needed to be validated in future exploration.

## MATERIALS AND METHODS

### Cell culture and treatment

Thyroid cancer cell line FTC-133, TPC-1, and BCPAP were purchased from American Type Culture Collection (MD, USA), cultured in a RPMI 1640 medium plus 10% FBS (Gibco, CA, USA) and 1% penicillin/streptomycin (Sigma, CA, USA). All cells were placed in a 37° C cell culturing incubator with humidified atmosphere containing 5% CO2. To obtain ^131^I-resistant cell lines (res-FTC-133 and re-TPC-1), cells were continuously exposed to gradually increased dose of ^131^I. To establish ^131^I -resistant TC cell models, FTC-133, TPC-1, and BCPAP cells were placed into 6-well plates and continuously exposed to stepwise generations of ^131^I (0.5 mCi) [17, 18]. After treatment for 12 h, IC50 of 131I radioactivity was analyzed by cell viability using MTT assay. The resistant cells were obtained after passaging for 8 generations.

For cell transfection experiments, miR-641 mimics, PTEN siRNA (siPTEN), EGOT siRNA (siEGOT) and their corresponding negative controls (NC), and EGOT overexpressing plasmid (pCMV-EGOT) were designed and synthesized by GenePharma (Shanghai, China). FTC-133 and TPC-1 were placed in 6-well plates at a density of 1 × 10^5^cells per well. The miRNA mimics, siRNAs, pCMV-EGOT plasmid or corresponding controls were mixed with 5 μl lipofectamine 3000 (Invitrogen, CA, USA) in Opti-MEM medium, left to stand for 20 minutes before added into culturing medium. Cells were collected for further experiments after transfection for 24 hours.

### Clinical samples

The paired primary thyroid cancer tissues (n = 36) who were undergoing surgical resection (with no treatment) were obtained from Hospital and approved by the cases for the investigation of this study. All patients were under diagnosis of pathologic detection.

### Cell viability

The viability of treated cells was determined by a cell counting kit (CCK8) (Beyotime, Shanghai, China). In brief, a total number of 5000 cells per well were placed in the 96-well plates and incubated in normal cell culturing condition. Twenty-four hours later, the 10 μL CCK-8 solution was added to each well and incubated for another 2 hours. Then, absorbance values at 450 nm were detected by a microplate reader (Sunrise, Tecan).

### Transwell assays

Transwell assays analyzed the impacts of bupivacaine on cell migration and invasion of cells by applying a Transwell plate (Corning, NY, USA) according to the manufacturer’s guidance. Briefly, the upper chambers were plated with around 1 × 10^5^ cells. Then solidified using paraformaldehyde (4%) and dyed using crystal violet. Invaded and migrated cells were recorded and calculated.

### Wound healing assay

The cells were put in the 24-well plates at 3 × 10^5^/well and cultured overnight to reach a full confluent as a monolayer. A 20μl pipette tip was applied to slowly cut a straight line across the well. Then the well was washed by PBS 3 times and changed with the serum-free medium and continued to culture. The wound healing percentage was calculated after 0 hours, 6 hours, and 12 hours by using the ImageJ software.

### Apoptosis

The transfected FTC-133, TPC-1, and BCPAP cells were digested and washed, suspended in 100 μL binding buffer and stained with Annexin V-FITC and PI staining reagents under the instruction of detection kit (Beyotime, China) in dark condition for 20 minutes. Afterward, the cells were washed and resuspended in binding buffer and measured by a flow cytometry (BD Biosciences, NJ, USA) immediately.

### Western blotting

FTC-133, TPC-1, and BCPAP cells received indicated treatment were washed in PBS and lysed with ice-cold lysis buffer containing a cocktail of proteinase inhibitors. Total protein was divided in SDS-PAGE gel and shifted to NC membranes. The membranes were soaked in fast blocking buffer for 15 minutes and incubated with specific primary antibodies, namely the anti-γ-H2AX (1:1000, Cell Signaling Technology, CST, MA, USA), anti-H2AX (1:1000, CST), anti-β-actin (1:2000, CST), anti-PTEN (1:1000, CST), at 4° C overnight. Next day, the membranes were incubated with corresponding HRP-conjugated secondary antibodies and ECL substrate (Beyotime, China). The visualization of proteins was performed by a Gel imaging system (BD Biosciences).

### RNA extraction and quantitative real-time PCR

FTC-133, TPC-1, and BCPAP cells were collected after indicated treatment, and total RNA was extracted using a PureLink RNA extraction kit (Thermo Fisher, MA, USA), and reversely transcribed to cDNA using a Reverse Transcription System Kit (Thermo Fisher). Evaluation of EGOT and PTEN cDNA levels was determined by a SYBR Mix kit (Takara, China) in a Real-time PCR system (BD Biosciences). For detection of miR-641, the RNA was processed by a miRNA cDNA first strand synthesis kit (Tiangen, Beijing, China) under the manufacturer’s protocol, and subjected to the SYBR Mix kit. The calculation of cDNA levels follows the 2^–ΔΔCt^ method. The GAPDH and U6 snRNA were adopted as internal control for the normalization. Primer sequences were as following: EGOT, F, 5’-CACTGCACAGGGAAACACAAA-3’, R, 5’-ACCCTGTTCATAAGCCCTGATG-3’; miR-641, F, 5’- TTATACTCTCACCATTTGGATC-3′, R, 5′- TGACAAGATTTTACATCAAGAA -3′; PTEN, F, 5’- TTTGAAGACCATAACCCACCAC -3’, R, 5’- ATTACACCAGTTCGTCCCTTTC-3’; U6, F, 5′-CTCGCTTCGGCAGCACATATACT-3′, R, 5′-ACGCTTCACGAATTTGCGTGTC-3′; GAPDH, F, 5′-CGGAGTCAACGGATTTGGTCGTAT -3′, R, 5′-AGCCTTCTCCATGGTGGT GAAGAC -3′.

### Dual-luciferase reporter gene assay

The wild-type sequence (WT) of EGOT and PTEN 3’UTR region which contain the binding site of miR-641 were accessed by using PCR amplification. The mutated fragments (Mut) of EGOT and PTEN 3’UTR were obtained by site-directed mutagenesis. All sequences were cloned into a psiCHECKTM-2 vector (Promega, WI, USA). Cells were planted in a 24-well plate and transfected with corresponding WT or Mut, together with miR-641 mimics or NC under the administration of Lipofectamine 3000. The cells were lysed 48 hours after treatment, and luciferase activity was determined by a Dual luciferase assay kit (Promega).

### RNA pulldown

Cells transfected with biotin-labeled miR-641 (RiboBio, Guangzhou, China) were harvested 48 hours after transfection, and were lysed by specific lysate buffer (Thermo Fisher). The cell lysates were incubated with magnetic beads (Thermo Fisher) at 4° C for three hours. The beads were washed and subjected to qPCR to detect the enrichment of EGOT.

### *In vivo* tumor xenograft

SCID nude mice (5-weeks old) were ordered from Vital River Laboratory (China) and fed in a SPF environment. The mice were randomly divided into two groups, then ^131^I-resistant TPC-1 cells (1×10^6^/100μL/mouse) treated with EGOT overexpressing plasmid were hypodermically injected in the nude mice. The width and length of tumor, and the body weight of mice were measured at indicated time. Tumor volume was calculated by the formula: width (mm)^2^ × length (mm)/2. The mice were anesthetized to death when tumor size reached 1000 mm^3^, and the tumors were collected.

### Statistical analysis

Data was shown as means ± SD and analyzed by a SPSS software (version 17.0). The Statistical significance were defined by p < 0.05 in Student’s t test or one-way ANNOVA analysis. The experiments were performed in biological triplicates.

## Supplementary Material

Supplementary Figures
