# Supplementary material for: Long non-coding RNA EGOT is associated with 131iodine sensitivity and contributes to thyroid cancer progression by targeting miR-641/PTEN axis
Source: Aging (Albany NY). 2023 Nov 21;15(22):13542–57. doi: 10.18632/aging.205284 (PMC10713430; doi:10.18632/aging.205284)
Supplement: Supplementary Figures [file aging-15-205284-s001.pdf]

SUPPLEMENTARY FIGURES

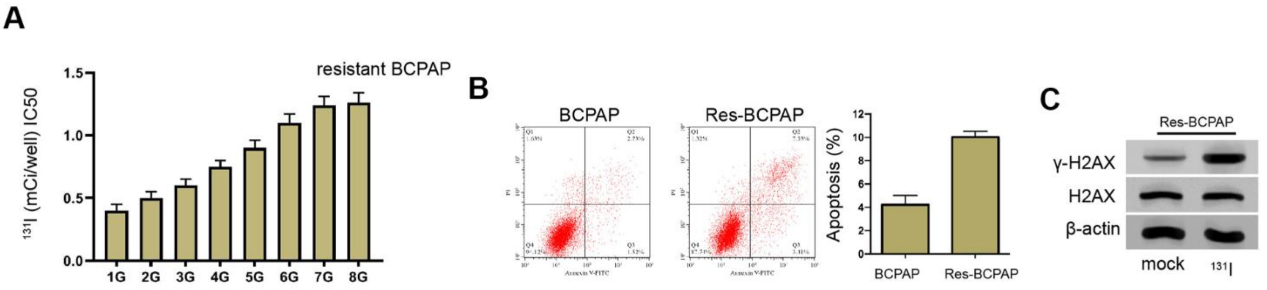

Supplementary Figure 1. The establishment of 131I-resistant TC cells. (**A**–**C**) BCPAP cells were treated with sub-lethal ^{131}I. (**A**) The ^{131}I -resistant BCPAP cells were established after 8-continuous passages. (**B**) Flow cytometry analysis of cell apoptosis in the cells. (**C**) Western blot analysis of γ-H2AX expression in the cells. mean ± SD, ** *P* < 0.01. Experiments were repeated at least biological triplicates.

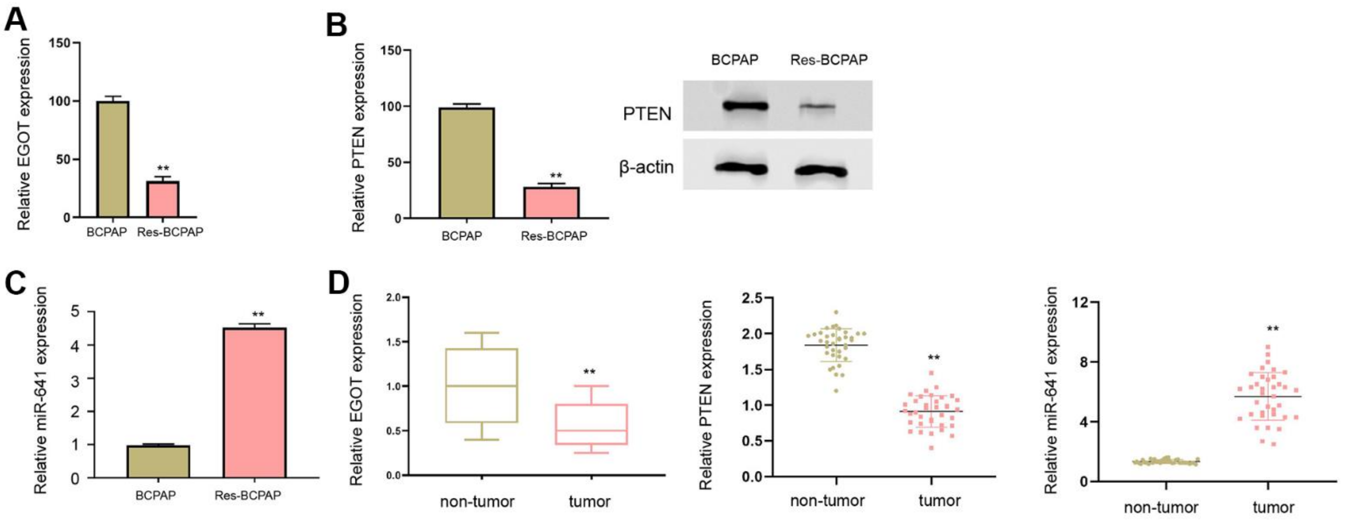

Supplementary Figure 2. EGOT and PTEN expression is decreased and miR-641 expression is increased in 131I-resistant TC cells. (**A**) The qPCR analysis of EGOT in ^{131}I -resistant BCPAP cells. (**B**) The qPCR and Western blot analysis of PTEN in ^{131}I -resistant BCPAP cells. (**C**) The qPCR analysis of miR-641 in ^{131}I -resistant BCPAP cells. (**D**) The qPCR analysis of EGOT, PTEN, and miR-641 in clinical tumor tissues and the adjacent normal tissues. mean ± SD, ** *P* < 0.01. Experiments were repeated at least biological triplicates.

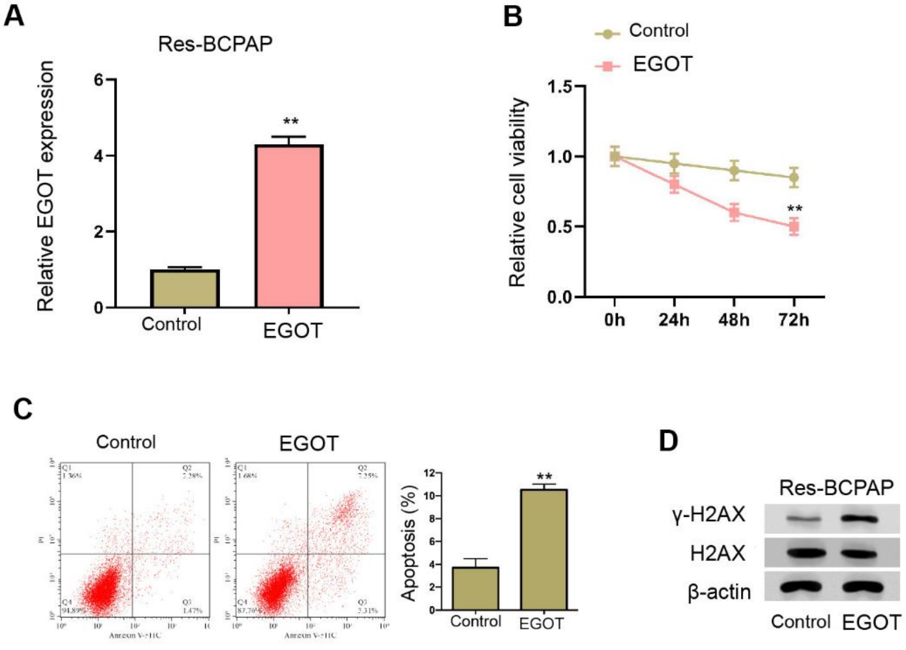

Supplementary Figure 3. EGOT represses viability, enhances apoptosis and induces DNA damage in 131I-resistant TC cells. (**A**–**D**) The ^{131}I -resistant and BCPAP cells were treated with EGOT overexpression vectors. (**A**) The qPCR analysis of EGOT in the cells. (**B**) CCK-8 analysis of cell viabilities. (**C**) Flow cytometry analysis of cell apoptosis in the cells. (**D**) Western blot analysis of γ-H2AX expression in the cells. mean ± SD, ** *P* < 0.01. Experiments were repeated at least biological triplicates.

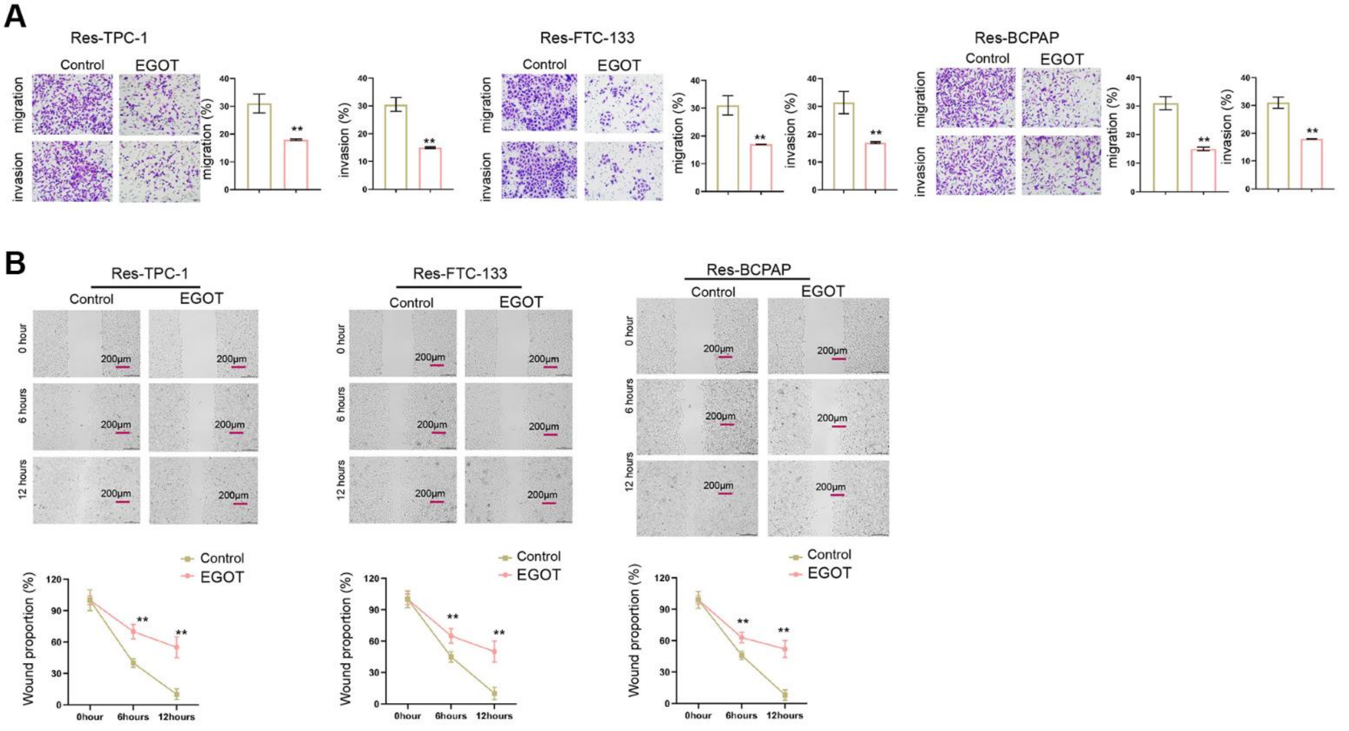

Supplementary Figure 4. EGOT represses migration and invasion in 131I-resistant TC cells. (**A**, **B**) The ^{131}I -resistant TPC-1, FTC-133, and BCPAP cells were treated with EGOT overexpression vectors. (**A**) The migration and invasion were measured by transwell in the cells. (**B**) The migration was analyzed by wound healing assays. mean ± SD, ** *P* < 0.01. Experiments were repeated at least biological triplicates.

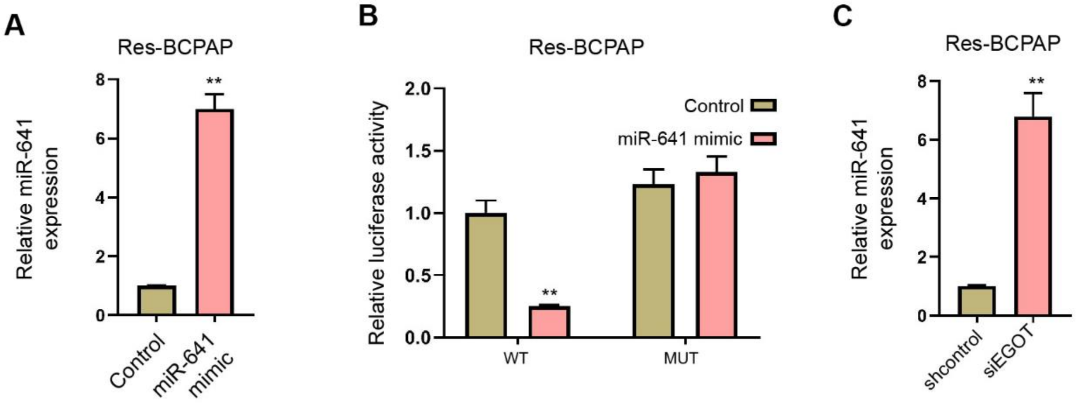

Supplementary Figure 5. EGOT is able to sponge miR-641 in 131I-resistant TC cells. (**A**, **B**) The ^{131}I -resistant BCPAP cells were treated with miR-641 mimic. (**A**) The qPCR analysis of miR-641 in the cells. (**B**) Luciferase reporter gene assays of EGOT luciferase activities. (**C**) The qPCR analysis of miR-641 in ^{131}I -resistant BCPAP cells treated with EGOT siRNA. mean ± SD, ** *P* < 0.01. Experiments were repeated at least biological triplicates.

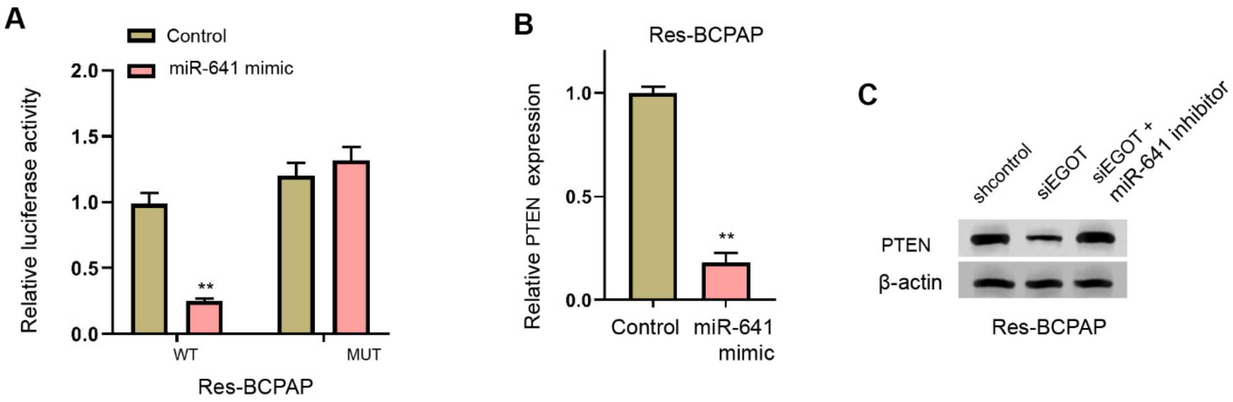

Supplementary Figure 6. MiR-641 can target PTEN in ^{131}I-resistant TC cells. (**A**, **B**) The ^{131}I -resistant BCPAP cells were treated with miR-641 mimic. (**A**) Luciferase reporter gene assays of PTEN mRNA 3'UTR luciferase activities. (**B**) The qPCR analysis of PTEN in the cells. (**C**) The Western blot analysis of miR-641 in ^{131}I -resistant BCPAP cells treated with EGOT siRNA, or co-treated with EGOT siRNA and miR-641 inhibitor. mean ± SD, ** *P* < 0.01. Experiments were repeated at least biological triplicates.

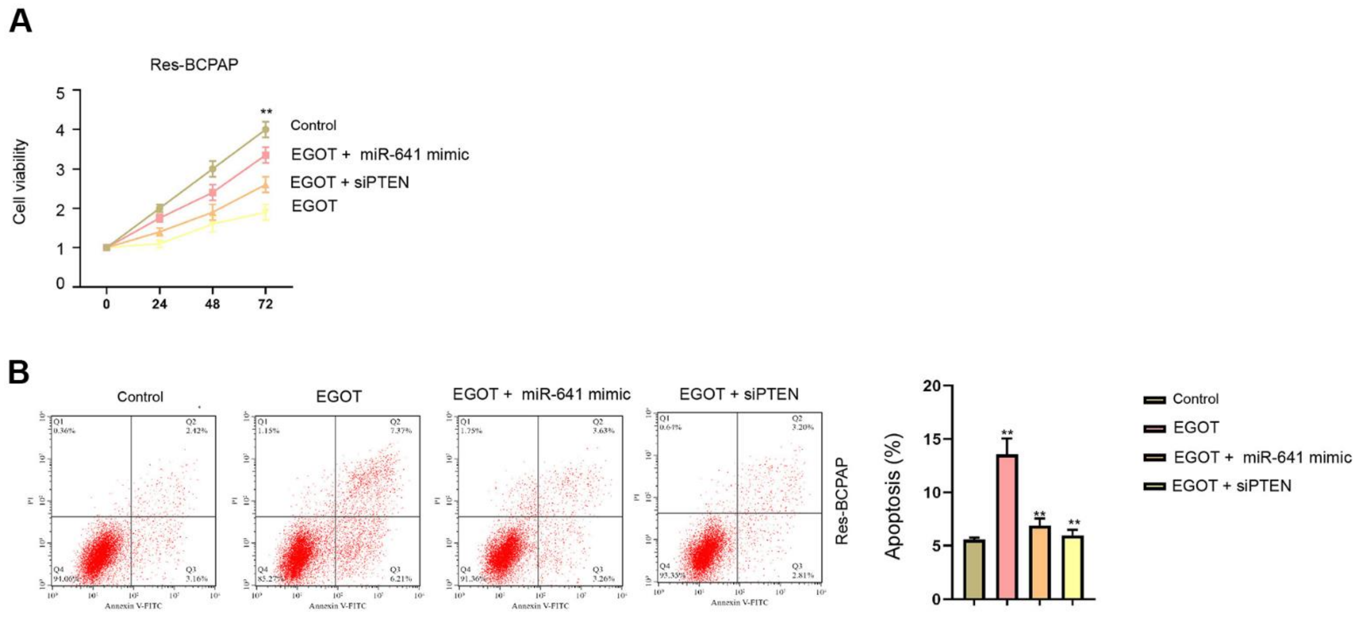

Supplementary Figure 7. EGOT regulates viability, apoptosis and DNA damage of 131I-resistant TC cells by targeting miR-641/PTEN axis. (**A**, **B**) The ^{131}I -resistant BCPAP cells were treated with EGOT overexpression vectors, or co-treated with EGOT overexpression vectors and PTEN siRNA or miR-641 mimic. (**A**) CCK-8 analysis of cell viabilities. (**B**) Flow cytometry analysis of cell apoptosis in the cells. mean ± SD, ** *P* < 0.01. Experiments were repeated at least biological triplicates.
